# Editorial: Extracellular matrix-like microenvironments for *in vitro* models and regenerative medicine

**DOI:** 10.3389/fbioe.2024.1505587

**Published:** 2024-10-14

**Authors:** Arturo Ibáñez-Fonseca, Alicia Fernández-Colino, Barbara Blanco-Fernandez, Dorela Doris Shuboni-Mulligan

**Affiliations:** ^1^ Lung Biology, Department of Experimental Medical Science, Lund University, Lund, Sweden; ^2^ Department of Biohybrid & Medical Textiles (BioTex), AME – Institute of Applied Medical Engineering, Helmholtz Institute, RWTH Aachen University, Aachen, Germany; ^3^ I+D Farma, Department of Pharmacology, Pharmacy and Pharmaceutical Technology, Faculty of Pharmacy and Institute of Materials (iMATUS), University of Santiago de Compostela, Santiago de Compostela, Spain; ^4^ Department of Biomedical and Translational Sciences, Eastern Virginia Medical School, Norfolk, VA, United States

**Keywords:** extracellular matrix, microenvironment, *in vitro* model, regenerative medicine, biomaterials, tissue engineering, decellularization, hydrogels

The extracellular matrix (ECM) is a key element of tissues and organs. It maintains their structure and provides biochemical and mechanobiological signals to the cells, having a prominent role in tissue homeostasis and regeneration (Karamanos et al., 2021). Given its importance, it is necessary to investigate novel ECM-like microenvironments to develop better *in vitro* models and to promote the regeneration of tissues where the ECM is impaired. With this Research Topic, we expected to open a window for current research addressing this need. We received 10 submissions, of which 7 were accepted for publication (Original research, n = 5; Perspective, n = 1; Review, n = 1).

The articles published in this Research Topic reflect the diversity of approaches used to bioengineer ECM substitutes. On the one hand, it is possible to use natural matrices, either keeping all the ECM components in decellularized tissue or selecting only one or a few to be used as biomaterials. For instance, Khan et al. offer an overview of the possibilities for repurposing biological waste from slaughterhouses to develop decellularized tissue scaffolds as natural ECM derivatives with use in xenotransplantation, helping to address the shortage of transplantable organs. They provide examples of different types of animal-derived xenografts, like blood vessels, kidneys, and tracheas, and describe several decellularization methods. The authors also highlight the challenges, including maintaining tissue compatibility and biocompatibility, and the importance of regulatory frameworks to support this innovative approach.

In another study, Isa et al. developed hydrogels made of type II collagen (COLII) from bovine cartilage and hyaluronic acid (HA), predominant components of the ECM in the nucleus pulposus (NP) of the intervertebral disc. The hydrogels showed an optimal swelling capacity and stability, with a sustained degradation over 14 days. Moreover, the authors used the HA/COLII hydrogels to encapsulate human Wharton jelly-derived mesenchymal stem cells, confirming their cytocompatibility. The cells cultured in the hydrogel also showed a higher expression of SOX9 mRNA (a NP cell marker) when exposed to transforming growth factor β-3, in comparison to the cells cultured in 2D. This result highlights the potential use of this type of natural ECM-like microenvironments to treat degenerative disc disease.

Two other articles explore the use of computational methods to model and evaluate various aspects related to the use of natural ECM derivatives. In this regard, Pantic et al. developed a platform for evaluating the effectiveness of corneal decellularization by looking at morphological changes in the stroma of the tissue. The authors combined traditional histological assessments with advanced image analysis techniques, including gray-level co-occurrence matrix and machine learning models like random forests and support vector machines. These methods showed high accuracy in identifying structural alterations when using various decellularization methods, offering a more objective and efficient approach to assessing scaffold quality for potential corneal transplantation applications.

As another computational method example involving the use of an ECM-like microenvironment, Ferre-Torres et al. developed an *in silico* model to simulate the chemotactic sprouting of endothelial cells through a fibrin-based hydrogel. They incorporated the effects of ECM structure and chemotactic factors like VEGF into a unified parameter in the model to successfully predict the dynamics of sprouting observed in microfluidic experiments. The study highlights the role of ECM remodeling and cellular interactions in angiogenesis, offering a novel predictive approach for understanding endothelial migration and matrix degradation during vascular development.

On the other hand, biomaterials that do not conform the natural ECM have also demonstrated their potential application to develop *in vitro* models and as tools in regenerative medicine. For instance, Kulka et al. describe the use of agarose/crystalline nanocellulose (CNC) composites for the 3D culture of bone marrow-derived mast cells (BMMC), as an improvement to regular 2D cultures. BMMCs maintained cell integrity, degranulation, and receptor expression when seeded inside the CNC/agarose microenvironment. However, the 3D culture inhibited the production of *de novo* synthesized mediators, including inflammatory cytokines like IL-1β and IL-6. The research demonstrates that CNC/agarose supports BMMC viability and certain immune functions but alters their inflammatory response, suggesting its potential use in controlling inflammation for therapeutic applications.

In another study, Smits et al. present a novel approach using dual photo-crosslinking of gelatin methacrylate (GelMA) hydrogels to dynamically modulate ECM stiffness. To evaluate the effect of changing stiffness on cells, the authors examined cardiac fibroblast activation and ECM-related gene expression. Their results show that fibroblasts cultured initially on softer substrates remained more quiescent even after stiffening, compared to those grown on stiff substrates from the beginning. The findings emphasize the importance of mechanical history in regulating cell behavior, offering insights into tissue remodeling and fibrosis, especially in post-myocardial infarction contexts. Importantly, this work highlights the possibility to engineer dynamic ECM-like microenvironments to mimic the spatiotemporal changes in the ECM.

Finally, Puertas-Bartolomé et al. review the use of elastin-like recombinamers (ELRs) for creating advanced *in vitro* models. This is a special type of biomaterial inspired in natural elastin that is not synthetic in a classical way, but that is genetically engineered and recombinantly produced. ELRs have similar properties than their natural counterpart in terms of mechanical properties, while offering tunable biological and physicochemical characteristics. The authors highlight the potential of ELRs in building scaffolds for 3D cell cultures, including spheroids, organoids and organ-on-a-chip models, and their use as bioinks for 3D bioprinting. They conclude that the versatility of the ELRs makes them ideal to build dynamic and biomimetic microenvironments for studying cell behavior, disease mechanisms, and drug responses.

During the last decades, the increasing knowledge of ECM composition and function in different tissues, together with research in areas like cell-ECM interaction and biomaterials science, has set the grounds for the achievement of promising approaches to mimic the native ECM (Han et al., 2020). Therefore, we are at an optimal point to imagine and generate new models and regenerative strategies that span the full range of tissues and organs in the body, as it is shown by the Research Topic of articles included in this Research Topic.

We would like to thank the authors for their contributions stressing the importance of developing advanced ECM-like microenvironments as more accurate *in vitro* models and more effective regenerative medicine strategies.
